# Transarterial Embolization and Percutaneous Ablation of Primary and Metastatic Soft Tissue Tumors

**DOI:** 10.3390/life13071485

**Published:** 2023-06-30

**Authors:** Chang Shu, Maria Lim, Adam Fang

**Affiliations:** 1 Post-Baccalaureate Premed Program, Goucher College, Baltimore, MD 21204, USA; chang.gavin.shu@gmail.com; 2 Department of Diagnostic Radiology and Nuclear Medicine, University of Maryland School of Medicine, Baltimore, MD 21201, USA; mlim@umm.edu; 3 Division of Vascular and Interventional Radiology, Department of Diagnostic Radiology and Nuclear Medicine, University of Maryland School of Medicine, Baltimore, MD 21201, USA

**Keywords:** interventional radiology, transarterial, percutaneous, embolization, ablation, tumors, sarcomas, soft tissue, TACE, cryoablation

## Abstract

Soft tissue tumors (STTs) include a range of benign and malignant tumors originating from soft tissues. Transarterial and percutaneous therapies are image-guided and minimally invasive approaches for managing primary and metastatic STTs. The objective of this review is to discuss transarterial and percutaneous therapies by examining the current literature, including indications, patient selection, safety, and effectiveness. Transarterial therapies (e.g., transarterial bland embolization and transarterial chemoembolization) involve the delivery of either embolic or chemotherapeutic particles using a catheter into arteries feeding the tumor, resulting in localized tumor destruction. Percutaneous therapies (e.g., radiofrequency ablation, cryoablation, irreversible electroporation, laser ablation, and magnetic resonance-guided high-intensity focused ultrasound) involve the delivery of either hot or cold temperatures, electrical current, laser, or ultrasound to specifically target tumor cells. Both therapies have been shown to be safe and effective for reducing morbidity and local control of STTs, specifically in patients who are surgically inoperable or who are unresponsive to conventional therapies. Accurate diagnosis, staging, and histological subtype identification are crucial for treatment selection. A multidisciplinary approach, a thorough understanding of tissue anatomy and surrounding structures, as well as individualized strategies based on assessment are essential for optimal patient care.

## 1. Introduction

Primary soft tissue tumors (STTs) are a group of benign and malignant tumors that arise from soft tissues. Examples of STTs are shown in Table 1. Benign STTs tend to grow slowly [1] and remain localized to the site of origin. While STTs can occur at any age, some subtypes have a tendency for specific age groups [2], with the highest prevalence in middle-aged and older adults. The most common type of benign STT is lipoma. Lipomas usually develop as soft, round, or oval lumps beneath the skin, most commonly in the neck, shoulders, back, or thighs. These STTs are typically painless, and they may grow to several centimeters in size. Neurofibromas and schwannomas are types of benign STTs that develop in the cells that protect and support the nervous system. Neurofibromas develop from cells that make up the outer sheath (epineurium) of peripheral nerves and mainly occur in adults between 20 and 40 years of age. Schwannomas arise from cells that produce the insulating myelin sheath around the nerve axons (Schwann cells) and can affect people of all ages. Abdominal wall endometriosis (AWE) and uterine fibroids (leiomyoma) affect the uterus and occur in females of childbearing age. While leiomyomas are smooth muscle tumors that grow from the uterine wall, AWE is a rare form of extra-pelvic endometriosis, with the majority of cases occurring after cesarean section or abdominal surgery [3,4]. Desmoid tumors, also known as aggressive fibromatosis, are a rare type of benign STT that arise from fibrous tissue. Desmoid tumors most commonly develop in the abdominal wall, and although these tumors are typically slow growing, they can be locally aggressive, invading nearby tissues and organs [5,6,7].

Malignant STTs, on the other hand, can grow rapidly, invade surrounding tissues, and metastasize to other parts of the body. Malignant STTs are composed of abnormal cells that often have atypical features, such as increased cell division, pleomorphism (variation in cell size and shape), or nuclear abnormalities [17]. The most common type of malignant STT in adults is liposarcoma [18,19,20], which arises from adipose tissue and can occur anywhere in the body where fat is present, but usually on the trunk, limbs, and retroperitoneum. Another common type of malignant STT is leiomyosarcoma [21,22,23], which arises from smooth muscle cells and accounts for 10 to 20% of all STTs. Leiomyosarcomas are commonly found in the uterus, abdomen, or pelvis in adults. Synovial sarcoma [24,25,26] arises from cells around the joint and tendon, occurring more commonly in young adults and accounts for 10% of all STTs. Other malignant STTs include gastrointestinal stromal tumors (GISTs), rhabdomyosarcoma, malignant peripheral nerve sheath tumors (MPNSTs), and retroperitoneal sarcomas [15,27,28].

Metastasis occurs when tumor cells spread to other parts of the body from the organ/tissue of origin [29,30]. Tumor cells break away from the primary tumor, travel through the blood or lymphatic system, and form a tumor deposit in another part of the body. Metastatic STTs are rare and can present as the initial manifestation of occult primary malignancy [31]. The most common sites of origin include the skin, lung, breast, kidney, colon and rectum, uterus, ovary, as well as head and neck [32]. Histologic or microscopic anatomical features of metastatic STTs can be easily mistaken for a variety of primary soft tissue sarcomas. As a result, routine immunohistochemical stains are recommended for defining the cell type and correct diagnosis, which is important for future treatment planning and prognosis [33]. 

The exact cause of STT is not well understood, but certain risk factors have been identified, including genetic syndromes and exposure to radiation and chemicals. Symptoms of STT vary depending on the type, location, and size of the tumor and may include palpable mass, pain, swelling, or restricted movement [34]. 

Treatment of primary STT typically involves a multidisciplinary approach, including surgery, radiation therapy, and systemic chemotherapy, depending on the size of the tumor, location, and aggressiveness [33]. Transarterial and percutaneous therapies provide an alternative approach to managing primary and metastatic STTs. These treatments offer minimally invasive options for targeted tumor destruction and palliation, with less morbidity compared with conventional surgical procedures [33].

## 2. Treatment of Primary and Metastatic STTs

### 2.1. Indications

Treatment of primary STTs varies depending on the location and stage of the tumor. Extensive and careful diagnostic tests must be performed before an effective and individualized treatment (palliative or radical) can be determined. Primary STT arises in soft tissues without any prior history of cancer and is most commonly present with symptoms of a palpable mass or swelling, which may be accompanied by pain or tenderness. In some cases, the tumor may compress adjacent structures, causing neurological or vascular symptoms [35]. For example, a sarcoma in the thigh may compress the femoral nerve, causing weakness or numbness in the leg. A retroperitoneal sarcoma may compress the ureter, leading to hydronephrosis and renal failure. 

One of the most common reasons for treating metastatic STT is symptomatic metastases, which cause pain, discomfort, and functional impairment. Treatment options commonly include surgery, radiation therapy, or systemic therapy, depending on the location and size of the metastases [33]. Another reason for the treatment of metastatic STT is potentially resectable metastases. In some cases, metastases may be isolated and limited in number, making them amenable to surgical resection. This approach may be appropriate for patients with good performance status and limited systemic disease burden.

The staging of STTs is based on the size and extent of the tumor, as well as the presence of metastasis. The most widely used staging system is the American Joint Committee on Cancer (AJCC) system, which assigns a stage from I to IV based on the tumor size, depth, and grade, as well as the presence of lymph node involvement and distant metastasis. Staging is important because it guides the choice of treatment and predicts the likelihood of recurrence and survival [36].

In addition to a patient’s age and tumor location, the histological subtype of the tumor is an important indicator of prognosis and treatment. There are over 50 subtypes of soft tissue sarcomas [37], each with distinct clinical and pathological features. Common subtypes include GIST, synovial sarcoma, myosarcoma, nerve tumors, liposarcoma, retroperitoneal sarcoma, and abdominal wall endometriosis. The histological subtype of the tumor can be determined by a biopsy, which is essential for the diagnosis and treatment of STT.

### 2.2. Patient Selection

The first step in the patient selection process [33] for transarterial or percutaneous treatments for primary, malignant, and metastatic STT is an accurate diagnosis and staging of the tumor. Imaging studies, such as computed tomography (CT), magnetic resonance imaging (MRI), or positron emission tomography (PET), help determine the size, location, and extent of the tumor, as well as any adjacent structures that may be at risk during the procedure [38,39]. The histological subtype and location of the tumor are also important for determining prognosis and directing the type of treatment. Tumors that are deep or inaccessible may be difficult to target with percutaneous ablation and may require surgery. Tumors that are close to critical structures, such as blood vessels, nerves, or organs, may require protective techniques, including gas dissection with air or carbon dioxide (CO_2_) and hydrodissection with 5% dextrose to ensure safe and effective treatment [40,41]. Furthermore, tumors smaller than 5 cm in diameter are more amenable to transarterial or percutaneous treatments than larger tumors which may require multiple treatments or a combination of treatments to achieve complete tumor shrinkage or destruction [33].

The patient’s overall health and medical history are important considerations in patient selection for transarterial or percutaneous treatments for primary, malignant, and metastatic STT. Patients’ prior medical complications and functional status, based on Eastern Cooperative Oncology Group (ECOG), are also important considerations for patient selection and for deciding their ability to tolerate the procedure [42].

## 3. Embolization Therapies

### 3.1. General Overview and Recommendations

The general principles of transarterial embolization for STT treatment involve blocking or reducing blood flow to the tumor, resulting in tumor shrinkage or destruction. This is achieved by injecting embolic agents into the arteries that supply blood to the tumor [43]. Embolization may be used alone or in combination with other treatments, such as surgery, radiation therapy, or systemic therapy [44]. 

A detailed understanding of the target tissue anatomy is critical to minimize non-target embolization. Image-guided techniques include fluoroscopy, CT, or cone-beam CT [38,45]. Fluoroscopy involves a real-time X-ray imaging technique, which is useful for guiding the catheter to the target site and for monitoring the injection of the embolic agent. In some cases, CT or cone-beam CT, in addition to the fluoroscopy, is useful for selectively guiding the catheter to the target tumor and providing detailed three-dimensional (3D) images of the vascular anatomy supplying the STT, which is valuable for preoperative planning [39].

**Figure 1:**
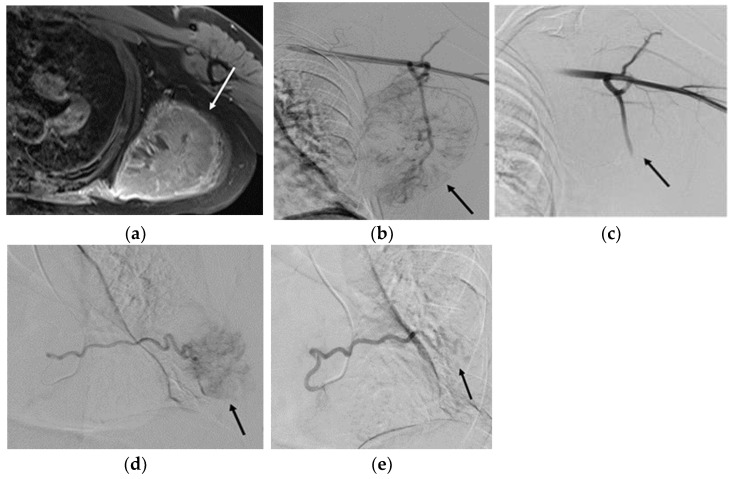
A 47-year-old male with a history of metastatic renal cell carcinoma with a left scapula soft tissue metastasis: (**a**) MRI of the left shoulder with contrast demonstrates a solid enhancing mass in the left scapula (arrow) measuring 8.8 × 9.2 × 9.3 cm with extension into the adjacent teres major and teres minor, subscapularis, and infraspinatus muscles with associated erosion of the inferior scapula. (**b**) Selective angiography of the left axillary artery demonstrates arterial supply via the lateral thoracic artery to the tumor (approximately 3/4 of the tumor volume). Gelfoam^®^ and particle embolization of the lateral thoracic artery were performed. (**c**) Post-embolization angiography of the lateral thoracic artery demonstrates satisfactory stasis and decreased tumor vascularity. (**d**) Selective angiography of the left seventh intercostal artery demonstrates arterial supply to the tumor (approximately 1/4 of the tumor volume). (**e**) Post-embolization angiography shows decreased tumor vascularity. MRI, magnetic resonance imaging.

During a typical transarterial embolization, a small incision is made, and a sheath (typically ranging from 4–6 French) is placed in the wrist or groin to access the arterial system. A catheter (typically ranging from 4–5 French) is inserted and advanced to an artery that supplies the tumor or abnormal tissue using image guidance. A microcatheter is advanced co-axially into the branch arteries supplying the tumor. Once the catheter is in place, embolic agents, such as particles and chemotherapy, are injected to block arterial blood flow to the tumor [46,47], as demonstrated in Figure 1.

Embolization procedures are performed under local anesthesia and sedation, and patients may need to stay in the hospital for several hours or overnight for observation. In general, patients can go home the same day or a day later [47]. After the procedure, patients may experience mild discomfort, such as pain or cramping in the treated area, and they may need to take pain medications for a few days. Some patients may also experience fatigue or flu-like symptoms, which typically resolve within a week [47].

### 3.2. Embolization Techniques

#### 3.2.1. Transarterial Bland Embolization (TAE)

TAE uses the above-described method of embolic agent delivery. Typical embolic agents include gelatin sponge, Gelfoam^®^ (Pfizer, New York, NY, USA), microspheres, polyvinyl alcohol (PVA) particles, or liquid embolic, such as Onyx^®^ Liquid Embolic System (Medtronic, Minneapolis, MN, USA) [48,49,50]. The procedure can be repeated if necessary. Delivering the embolic agents directly to the tumor minimizes the exposure of healthy tissues to the agents and reduces the risk of non-target side effects.

#### 3.2.2. Transarterial Chemoembolization (TACE)

TACE involves the additional step of injecting chemotherapy drugs directly into the blood vessels that supply the tumor, followed by the injection of particles to block the blood flow to the tumor [42]. Commonly used chemotherapeutic drugs in TACE include cell growth inhibitors doxorubicin and cisplatin and the potent deoxyribonucleic acid (DNA) crosslinker mitomycin C [51]. While TACE utilizes similar embolic agents as TAE, such as gelatin sponge and microspheres, this therapy also uses additional agents, including lipiodol and drug-eluting beads (DEBs) [51]. This combination of chemotherapy and embolization is designed to attack the cancer cells and deprive them of the nutrients and oxygen they need to grow. The ability to deliver the mixture of chemotherapy drugs directly to the tumor minimizes damage to healthy tissue.

### 3.3. Outcomes in Embolization of Primary and Metastatic STT

While there is still limited research on the use of TAE treatments for STTs, early studies have shown promising results in reducing tumor size and alleviating pain. A retrospective study conducted by Nagata et al. [52] evaluated the outcomes of TAE for primary and metastatic osseous and soft tissue sarcomas in 38 patients. The study reported 79% of cases (8 of 9 with primary tumors, and 22 of 29 with metastases) with significant tumor necrosis and a size reduction in 56% of the tumors after the first 3 months. Pain control 1 week after the procedure was experienced in 8 of 9 patients with painful primary tumors. The 1-year survival rate was 38.1%, the median survival was 18 months, and the longest survival was 84 months [52].

Shimohira et al. [53] investigated the feasibility and safety of preoperative TAE with a gelatin sponge for embolizing hypervascular bone and STT to reduce intraoperative blood loss. The technical success rate was 100%, with a clinical success rate of 89%. The use of analgesic drugs was sufficient in controlling the local pain experienced by 13 out of the 37 patients. 

Ni et al. [54] showed in 10 patients that DEB-TACE was safe and effective for patients with STT refractory to systemic chemotherapy. The objective response rate was 30%, with a median progression-free survival of 9.5 months (range of 2 to 15 months), and the median survival time was 21 months (range of 11 to 30 months). The overall survival rate at 1 and 2 years was 90% and 30%, respectively [54].

Jiang et al. [55] investigated the use of TACE in treating 39 patients with unresectable STT. Jiang et al. concluded that TACE could be an effective treatment for advanced STT. The mean overall survival was 23.7 ± 2.1 months, with 1-year OS of 71.5%, 2-year OS of 45.8%, and 3-year OS of 32.5% post-treatment. When compared to chemoinfusion, TACE treatment also led to lower cancer pain evaluated by visual analog scores (VAS) and longer relapse intervals.

Kim et al. [56] demonstrated that of 11 patients treated with TACE as an alternative local treatment for desmoid tumors, 10 patients (90.9%) showed a reduction in overall tumor volume, partial to near-complete tumor necrosis, maximum VAS, and continued volume reduction in residual tumors. Based on these findings, Kim et al. concluded that TACE may be used as a safe and effective local treatment alternative for patients with desmoid tumors.

Lastly, Elnekave et al. [57] focused on patients with locally aggressive desmoid fibromatoses. After a median follow-up period of 8 months (interquartile range, 3–13), the study found that median tumor volumes decreased by 59% (interquartile range, 40–71%), indicating a reduction in size and a decrease in tumor cellularity. Of 24 patients, 9 (39%) demonstrated a partial response, and 12 (52%) had stable disease. Elnekave et al. inferred that TACE could be safely considered in treating desmoid fibromatoses.

## 4. Ablation Therapies

### 4.1. General Overview and Recommendations

In recent years, percutaneous ablation has emerged as an alternative to surgical resection, particularly for patients, who are not surgical candidates due to the location or size of their tumor, or for patients who wish to avoid the risks and complications associated with surgery. Percutaneous ablative techniques include chemical ablation, thermal ablation (e.g., cryoablation, radiofrequency ablation [RFA], microwave ablation (MWA), and lasers), and non-thermal ablation (e.g., magnetic resonance-guided high intensity focused ultrasound [MR-guided HIFU] and irreversible electroporation [IRE]) [58]. These techniques offer several advantages over surgery, including a lower risk of complications, reduced hospitalization time, and faster recovery. Additionally, percutaneous ablation may be repeated if needed, making it a viable option for patients with recurrent primary or metastatic disease. For lesions <3 cm, ablations can provide curative treatment [58]. 

When using ablative techniques to treat STTs, a complete evaluation of the surrounding anatomy and tissue is necessary. Vital structures, such as nerves and blood vessels, may impact patient selection and require additional or even specialized protection and monitoring techniques. Thermal sink effects should be considered in selected patients with lesions near major blood vessels. To ensure safety, thermocouples for temperature monitoring and motor evoked potentials, electromyography (EMG), and electroencephalogram (EEG) may be utilized to minimize nerve damage. Skin protection, hydrodissection, CO_2_, or air insufflation are techniques that can be used to provide thermal protection [58]. Furthermore, the targeted lesion must be easily accessible percutaneously and far enough away from vital structures. The necessary margin of safety is dependent on the interventional radiologist’s ability to visualize, displace, and monitor adjacent critical structures. During tumor ablation, the intraprocedural discomfort of patients is managed using local anesthesia, moderate intravenous sedation, or general anesthesia. 

**Figure 2:**
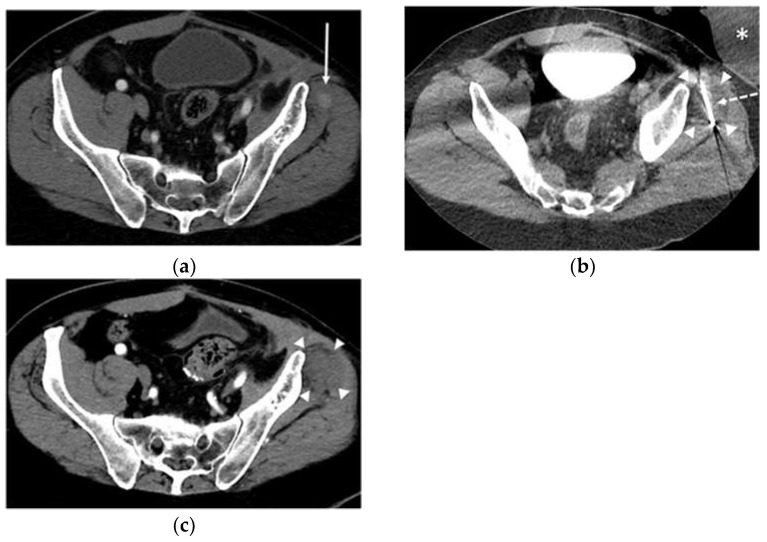
A 61-year-old male with metastatic high-grade undifferentiated pleomorphic sarcoma of left gluteus medius muscle: (**a**) Contrast-enhanced axial CT image shows a 1.2 cm enhancing soft tissue mass (arrow) in the left gluteus medius muscle compatible with distant metastatic disease. (**b**) Axial CT image acquired at 5 min into the freeze cycle during cryoablation procedure shows one of the three probes (dashed arrow) located across the mass and developing ice ball (arrowheads). Warm-saline bag (asterisk) was placed over the skin for protection during cryoablation. (**c**) Contrast-enhanced axial CT image 3 months after cryoablation demonstrated post-ablation changes of the left gluteus medius muscle (arrowheads) without residual or recurrent disease. CT, computed tomography.

Image guidance plays an integral role in percutaneous ablative therapies. While ultrasound is an excellent option for visualizing STTs in real-time, its use is limited to relatively superficial masses. MRI offers superior contrast resolution, real-time image guidance, and possible temperature monitoring. CT is commonly used for providing rapid and precise placement of devices using CT-fluoroscopy and near real-time monitoring of cryoablation with non-contrast CT imaging [38,39], as demonstrated in Figure 2.

### 4.2. Ablation Techniques

#### 4.2.1. Cryoablation

Cryoablation produces extremely cold temperatures to provide lethal ablation to target tissue using a combination of argon and helium gas [58]. Cryoprobes can be placed percutaneously directly into the tumor. A typical ablation cycle involves 2 cycles of freezing for 10 min, separated by cycles of active thawing for 5 min [33]. Visualization of the ice ball under image guidance allows active monitoring to avoid damage to critical structures. Due to the anesthetic effects of freezing, cryoablation has the benefit of decreased peri- and post-procedural pain.

#### 4.2.2. RFA

RFA utilizes high-frequency electrodes with alternating currents placed percutaneously within a target tissue. Frictional heat (60–100 °C) is generated due to ionic agitation and ultimately results in protein denaturation and coagulation necrosis [58]. The temperature and duration produced by the radiofrequency waves can be adjusted during the procedure.

#### 4.2.3. MWA

MWA uses electromagnetic microwave energy to induce an ultra-high frequency (915 MHz or 2450 MHz) alternating electric field. This causes agitation of water molecules, which produces frictional heat (60–100 °C) and leads to thermal coagulation and local tissue necrosis [58].

#### 4.2.4. Percutaneous Laser Ablation (PLA)

PLA involves the use of diode lasers, neodymium yttrium aluminum garnet (Nd:YAG) lasers, and CO_2_ lasers [58,59]. Diode lasers emit infrared light and are used for the ablation of tissue. This laser is used to treat smaller STTs, particularly those located near the skin surface. Nd:YAG lasers emit high-energy light that can penetrate deep into the tissue, making them effective for treating STTs located deep within the body. CO_2_ lasers emit light absorbed by water, leading to rapid heating and destruction of the targeted tissue. This laser is often used for superficial STTs because it is precise and effective for vaporizing tissue. PLA is optimal for treating small lesions in critical structures due to the use of small caliber applicators and the extreme precision of the ablation area.

#### 4.2.5. MR-Guided HIFU

MR-guided HIFU involves the use of focused ultrasound energy delivered with MR guidance to create focal elevated temperatures at the targeted lesion. An ultrasound transducer located in the MR table is housed in an oil bath and is coupled to the patient’s skin via a moistened gel pad. The patient is placed on the MR table, and the targeted lesion is aligned with the transducer [58]. Pre-treatment planning images are obtained in axial, coronal, and sagittal planes with and without fat suppression to determine the appropriate energy needed to be delivered to the target tissue. After verification of the corrected targeted area using low subtherapeutic sonication, full therapeutic power is applied with a focused ultrasound phased-array system for treatment.

#### 4.2.6. Irreversible Electroporation (IRE)

IRE is a non-thermal ablative modality using percutaneously placed fine antennas across a target area to produce direct current (25–45 A) electrical pulses of high voltage (1500–3000 V) [58]. A strong external electric field is created that induces the formation of nano-sized pores in the cell membrane resulting in cell death via apoptosis [60]. IRE minimizes damage to the surrounding healthy tissue, including collagen and other supporting structures.

### 4.3. Outcomes in Ablation of Primary and Metastatic STT

Although the use of percutaneous ablation treatments for primary STT is relatively novel, a growing body of research has investigated their effectiveness in reducing tumor size, alleviating pain, and improving quality of life. Early studies have shown promising results. Ablation techniques have been increasingly used in treating metastatic soft tissue sarcomas, and several reviews and individual studies have reported on their use. In addition to the effectiveness of ablation treatments for metastatic soft tissue sarcomas, studies were performed looking into the safety and effectiveness of ablation treatments in concert with other treatments.

A study by Fan et al. [61] evaluated the outcomes of percutaneous cryoablation for recurrent STT in 39 patients. The study reported immediate pain relief in 57.1% in the small-tumor group (<10 cm) and delayed pain relief in 77.3% in the large-tumor group (≥10 cm). The study also reported an overall survival of 67% after 13.4 months.

Doshi et al. [62] examined the safety and feasibility of cryoablation during immunotherapy in 16 patients with metastatic STT. The study involved cryoablation of 34 tumors, with liposarcoma being the most common tumor subtype, and 81% of patients demonstrating progression after multiple lines of chemotherapy. Complete response was observed in all tumors cryoablated with complete intention, and clinical benefits (complete response, partial response, and stable disease) were observed in seven patients. The median overall survival was 14.1 months, and the median PFS was 2.3 months. The study concluded that cryoablation is safe and feasible for patients with metastatic STT undergoing immunotherapy.

Susa et al. [63] reported analgesic efficacy and local tumor control using CT-guided cryoablation treatment for locally recurrent or metastatic bone and STTs. The benefits of percutaneous cryoablation are the potential for improved local control, analgesic efficacy, and reduced complication [64].

Yamakado et al. [65] evaluated the outcomes of RFA for the treatment of recurrent STT in 52 patients. At the end of the follow-up (mean follow-up, 25.5 ± 24.2 months), 28.8% (15/52) of patients were tumor-free. Residual tumors were found in the remaining 59.6% (31/52) of patients. The overall survival rates were 73.4% at 1 year, 39.3% at 3 years, and 34.3% at 5 years in all patients. Lastly, the study reported a low rate of major complications (0.9%).

Between the two studies by Aubry et al. [66] and Kastler et al. [67], 11 patients with STTs underwent MWA treatment. Aubry et al. reported success rates (≥80% necrosis) of 80% at 3 months, 76.9% at 6 months, and 63.6% at 12 months. Kastler et al. compared the preprocedural mean VAS with the postprocedural VAS 6 months after MWA treatment and observed an overall reduction in approximately 5 out of 10 on the VAS. Both studies concluded that MWA is a safe, effective, and efficient treatment for malignant STT. Overall, percutaneous ablation treatments can provide significant pain relief and improve the quality of life for patients with primary and metastatic STT. 

There are limited case reports with small sample sizes for laser ablation, IRE, and MR-guided HIFU therapies. However, Jiang et al. [68] and Angelis et al. [69] demonstrated favorable and promising outcomes for treating metastatic and recurrent STT using percutaneous Nd:YAG laser ablation on smaller patient samples. Another study by Ghanouni et al. concluded that MR-guided HIFU might be an appropriate first-line treatment for locally aggressive tumors where standard therapies may be ineffective or dangerous [70]. Lastly, a study by Martin et al. elucidated that IRE, combined with other multidisciplinary therapies, can achieve high local disease control and long local recurrence-free survival (LRFS) [71].

## 5. Conclusions

In conclusion, transarterial and percutaneous therapies are valuable treatment options for primary and metastatic STTs. While surgery, radiation therapy, and systemic chemotherapy have traditionally been the mainstays of treatment for these tumors, the minimally invasive nature of transarterial and percutaneous therapies provides numerous benefits, including reduced morbidity and improved patient outcomes. TAE and TACE have been shown to be safe and effective treatments in reducing tumor size and pain in patients with STT. Percutaneous thermal ablation techniques, including RFA, MWA, and cryoablation, have demonstrated improved palliation and local control of STT. Although further research is needed to determine the optimal treatment approach for individual patients, these emerging therapies offer hope for improved outcomes and quality of life for patients with primary and metastatic STT.

## Figures and Tables

**Table 1:** Examples of different STT types and details for each type.

Tumor Subtype		Benign or Malignant	Incidence (per 100,000 per Year)	Age	Origin	Location
GIST (Gastrointestinal Stromal Tumor) [8]		Malignant	1–1.5	Adults— 55′s to 65′s	Connective tissue cells in digestive tract	Most common in the stomach (50–70%) Small intestine (20–30%)
Synovial sarcoma [9]		Malignant	0.81–1.42	Adolescents and young adults	Primitive mesenchymal cells	Occurs anywhere in the body (10% of all STTs) Majority arises in extremities, adjacent to the knee joints
Myosarcoma	Leiomyosarcoma [10]	Malignant	1.0	Adults— any age	Smooth muscle cells	Most commonly found in the uterus, abdomen, or pelvis (10–20% of all STTs)
	Rhabdomyosarcoma [11]	Malignant	0.44	Children and adolescents	Skeletal or voluntary muscle cells	Majority arises in the extremities. Very infrequently in the head and neck regions
Nerve tumors	Neurofibroma/ Schwannoma [12]	Benign	1.09–1.2	Adults— 20′s to 40′s (Neurofibroma) Any age (Schwannoma)	Cells that protect and support the nervous system e.g., Schwann and mast cells	Soft bumps on or under the skin (Neurofibroma) Occurs anywhere in the body with Schwann cells (Schwannoma)
	Malignant peripheral nerve sheath tumor (MPNST) [13]	Malignant	0.2	Adults— young to middle aged	Cells that cover and protects the peripheral nerves	Occurs anywhere in the body Majority arises in the deep tissues of the arms, legs, and the trunk
Liposarcoma [14]		Malignant	0.4–1.1	Adults– 35′s to 65′s	Adipose and fatty tissue cells	Occurs anywhere in the body Majority arises on the trunk, limbs, and the retroperitoneum
Retroperitoneal Sarcoma [15]		Malignant	0.3–0.4	Adult men— 60′s	Soft tissue Sarcomas	Originates in the retroperitoneum
Abdominal wall Endometriosis (AWE) [16]		Benign	2.57 (~1% C-sections per year)	Female— childbearing age	Endometrial cells	Extra-pelvic endometriosis in the abdominal wall (following C-section or pelvic surgery)

C-section—Cesarian section; STT—soft tissue tumor.

## Data Availability

Not applicable.
